# ‘Sono-cardiopulmonary resuscitation’ in COVID-19: a proposed algorithm

**DOI:** 10.1136/postgradmedj-2020-138747

**Published:** 2020-09-17

**Authors:** Brunda RL, Vishakh C Keri, Tej Prakash Sinha, Sanjeev Bhoi, Prakash Ranjan Mishra

**Affiliations:** Emergency Medicine, All India Institute of Medical Sciences, New Delhi, India; Infectious Diseases (Medicine and Microbiology), All India Institute of Medical Sciences, New Delhi, India; Emergency Medicine, All India Institute of Medical Sciences, New Delhi, India; Emergency Medicine, All India Institute of Medical Sciences, New Delhi, India; Emergency Medicine, All India Institute of Medical Sciences, New Delhi, India

## INTRODUCTION

As cardiac arrest occurs in around 20% of the patients with severe COVID-19, a large number of them will require immediate resuscitative efforts.^1^ Cardiopulmonary resuscitation (CPR) in COVID-19 pandemic has become a source of speculation and debate worldwide. Healthcare professionals (HCPs) resuscitating this subset of patients are subject to fears and enormous mental stress pertaining to risk of transmission, breach in personal protective equipment (PPE), unsure effectiveness of PPE and nevertheless bleak positive outcomes in patients despite best resuscitative measures.^2^ CPR, which is conventionally deemed to be life-saving for patients, appears as an aerosol-generating procedure risking lives of HCPs caring for patients with COVID-19. Protected code blue algorithm has been formulated to address both performer and patient safety.^3^

## POCUS-INTEGRATED CPR: WHY THE NEED IN COVID-19?

Danilo Buonsenso and colleagues have described COVID-19 era as demanding less stethoscope and more ultrasound usage in clinical practice.^4^ PPE is now an essential measure for HCP protection, and goggles used as a part of PPE are associated with fogging and poor visibility. This coupled with the inability to confirm endotracheal tube position with stethoscope due to poor accessibility in PPE, increases the risk of oesophageal intubation, re-intubation attempts, aerosol generation and thus HCP exposure. Bedside ultrasound could act as visual stethoscope in the described scenario. Sono-CPR in COVID-19 can help intervene quickly in treatable cases and reduce the time spent by HCP in futile resuscitative efforts. Reduced time spent equates to reduced duration of aerosol exposure and thus reduced risk of transmission. Various algorithms are described for sono-cardiopulmonary resuscitation (sono-CPR) during cardiac arrest, but none are discussed to address patients with COVID-19.^5^ It would hence be wise to integrate bedside point-of-care ultrasound (POCUS) in the code blue algorithm.

## HOW THE BEDSIDE TOOL HELPS?

Hypoxemia and respiratory failure attribute over 80% aetiology of cardiac arrest in patients with COVID-19.^1^ Prioritising oxygenation and ventilation using definitive airway and use of high-efficiency particulate air filters reduces airborne transmission, thereby making early intubation the dictum of resuscitation.^3^ Considering poor visualisation due to fogging with the goggles and face shield, inability to use stethoscope and lack of availability of end-tidal CO2 (EtCO2) in resource constraint settings, ultrasound-guided real-time intubation by trained HCP or endotracheal tube (ETT) placement confirmation post intubation could prove beneficial. Confirming ETT placement and direct visualisation of oesophagal lumen can be done using a linear ultrasound probe.^6^ In cases of oesophageal intubation, tissue-air hyperechoic lines are visualised in both trachea and oesophagus, referred to as ‘double-track sign’.

State of hypercoagulability and myocardial dysfunction exist in patients with COVID-19, hence increasing the likelihood of myocardial infarction or pulmonary thromboembolism as aetiologies of cardiac arrest.^7^ Regional wall motion abnormality, dilated right atrium or right ventricle, plethoric inferior vena cava are easily identified by goal-directed echocardiography. Pneumothorax has been reported in patients with COVID-19, and ultrasound can identify absence of lung sliding, helping in quick needle thoracocentesis in arrest and peri-arrest cases. Few cases of cardiac tamponade owing to myopericarditis have also been reported and bedside ultrasound can help diagnose and perform pericardiocentesis in such patients.

Literature suggests that the chances of Return Of Spontaneous Circulation (ROSC) and survival to hospital admission at 24 hours is better in patients with baseline cardiac activity rather than no baseline cardiac activity. In patients with no baseline cardiac activity on arrival, one can withhold CPR, thereby protecting the HCP in this resource-intensive, aerosol-generating futile resuscitative effort.^8^ Asystole could be the disguised entity of fine ventricular fibrillation, which can be confirmed by fibrillatory cardiac activity on transthoracic echocardiography and can be defibrillated, thereby increasing the chances of earlier ROSC.^9^

## POCUS-INTEGRATED CPR: THE PROPOSED ALGORITHM

**Figure 1:**
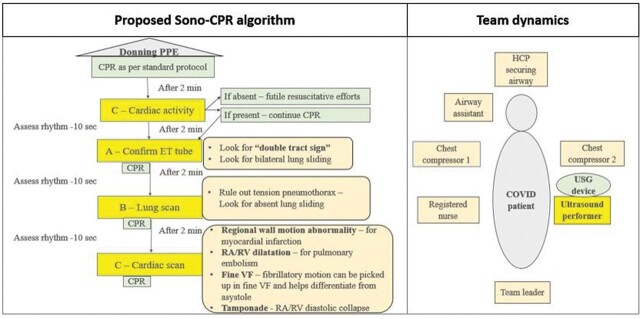
Proposed algorithm for integration of POCUS during CPR in patients with COVID-19 with team dynamics. The illustration is original work of the authors Dr Brunda RL and colleagues. CPR, cardiopulmonary resuscitation; HCP, healthcare professional; POCUS, point-of-care ultrasound; PPE, personal protective equipment; RA, right atrium; RV, right ventricle; VF, ventricular fibrillation; USG, ultrasonography.

CPR is a chaotic scenario, and to prevent added chaos, there is a need for a well-trained ultrasound performer placed in an appropriate area (figure 1). Intubating room needs to consist of minimal necessary number of HCPs, and all of them should be equipped with full PPE. Ultrasound device could be a potential fomite facilitating cross-transmission and requires adequate protection of machine and its components with a transparent cover, sheet or bag. When unavailable, low-level disinfectant solution should be used between each patient.

When a patient experiences cardiac arrest, there is a need for HCPs with full PPE to check pulse and begin CPR as per standard guidelines. After 2 min of CPR, if there is no ROSC, during the 10 second pause for rhythm assessment, a trained HCP can perform POCUS in a stepwise manner. Each step needs to be performed individually during 10 second pause without prolonging delay between chest compressions and compromising the quality of CPR. Any treatable aetiology identified during the algorithm requires immediate intervention.

Step 1: *Assess cardiac activity*—Sub-xiphoid view can be procured and cardiac activity assessed. If absent, consider termination of efforts, and if present, resuscitative efforts can be continued.

After repeating 2 min cycle of CPR, if there has been no ROSC, consider hypoxic aetiology as the cause of arrest in patients with COVID-19 and intubate without delay. Withholding chest compressions during intubation is recommended.^3^

Step 2: *Assess ETT placement*—At the level of thyroid gland, above the suprasternal notch, place ultrasound probe transversely and visualise the oesophagus.^10^ If the posterior wall of oesophagus is obscured by a dark acoustic shadow or if there is ‘double-track’ sign, consider failed endotracheal intubation and perform immediate re-intubation.

Step 3: *Assess lung for pneumothorax*—Assess lung sliding, and if absent look for ‘stratosphere sign’ in M-mode of ultrasound.^10^ If detected, perform immediate needle thoracocentesis.

Step 4: *Assess for Cardiac etiology of arrest*—Obtain sub-xiphoid window preferably, and look for the presence of cardiac tamponade, chamber dilatation or collapse, regional wall motion abnormality and cardiac contractility.

Availability of trained personnel and smaller portable ultrasound devices makes its use during cardiac arrest plausible.

CPR with the help of POCUS could thus prove to improve chances of ROSC and also reduced infection transmission to HCP by early identification, treatment of reversible causes and avoidance of prolonged efforts. Sono-CPR appears to be more HCP-friendly than prolonged blind CPR and necessitates its utility in the era of COVID-19 addressing performer safety as well as patient safety.
